# High-Speed Fluorescence Imaging Corroborates Biological Data on the Influence of Different Nozzle Types on Cell Spray Viability and Formation

**DOI:** 10.3390/jfb15050126

**Published:** 2024-05-14

**Authors:** Miriam Heuer, Mehdi Stiti, Volker Eras, Julia Scholz, Norus Ahmed, Edouard Berrocal, Jan C. Brune

**Affiliations:** 1 German Institute for Cell and Tissue Replacement (DIZG, gemeinnützige GmbH), Haus 42, Köpenicker Str. 325, 12555 Berlin, Germany; 2 Division of Combustion Physics, Department of Physics, Lund University, P.O. Box 118, 22100 Lund, Sweden; 3 Institut de Mécanique des Fluides de Toulouse (IMFT), CNRS, Université de Toulouse, 31400 Toulouse, France

**Keywords:** keratinocytes, cell spray, high-speed imaging, PDA droplet sizing, burns, nozzles

## Abstract

Treating severe dermal disruptions often presents significant challenges. Recent advancements have explored biological cell sprays as a promising treatment, but their success hinges on efficient cell delivery and complete wound coverage. This requires a good spray distribution with a small droplet size, high particle number, and ample surface coverage. The type of nozzle used with the spray device can impact these parameters. To evaluate the influence of different nozzles on spray characteristics, we compared air-assisted and unassisted nozzles. The unassisted nozzle displayed small particle size, high particle number, good overall coverage, high cell viability, preserved cell metabolic activity, and low cytotoxicity. Air-assisted nozzles did not perform well regarding cell viability and metabolic activity. Flow visualization analysis comparing two different unassisted nozzles using high-speed imaging (100 kHz frame rate) revealed a tulip-shaped spray pattern, indicating optimal spray distribution. High-speed imaging showed differences between the unassisted nozzles. One unassisted nozzle displayed a bi-modal distribution of the droplet diameter while the other unassisted nozzle displayed a mono-modal distribution. These findings demonstrate the critical role of nozzle selection in successful cell delivery. A high-quality, certified nozzle manufactured for human application omits the need for an air-assisted nozzle and provides a simple system to use with similar or better performance characteristics than those of an air-assisted system.

## 1. Introduction

Burn injuries pose a significant health threat for individuals and successful treatment of such injuries is crucial for tissue reconstruction. In 2022, the DGV burn register reported 2504 burn cases, with 1787 of the cases involving children [1]. These injuries can be caused by heat [2], chemicals [3], or electricity [4], and patient treatment methods are constantly being improved. High mortality is observed in patients with complicated wounds, particularly in elderly patients with extensive surface area burns [5]. These injuries can lead to impaired skin function depending on the severity of the burn. Type IIb and III burns necessitate wound closure and re-epithelialization to restore skin function and minimize scar formation [6]. Autografting is a commonly used treatment technique, but is reliant on donor skin availability. Skin replacement or skin substitutes can be used to cover the wound bed following a burn injury [7]. Skin replacements are available in the form of autografts or allografts. Skin substitutes, on the other hand, are a mix of cells or tissues, and can include engineered tissue [8]. Autografts involve harvesting the patient’s own healthy skin and can be either full-thickness skin grafts (FTSGs) or split-thickness skin grafts (STSGs). The use of the patient’s own skin to cover the wound bed can lead to harvest site infections and the need for a secondary surgical site. Another method used is autologous cell culture, which provides the option to culture and expand the patient’s skin [9,10], unlike autografting, which does not increase cells or tissue material. Thus, the use of autologous cell culture is suggested in situations where there is a lack of donor material or when the skin is unsuitable for treatment purposes. The surgical priority is to sufficiently close the wound [11] and increase patient cell material through autologous cell culture [12].

Keratinocytes are the primary cell type involved in wound healing and these epithelial cells are essential in the proliferative phase of wound healing [13]. Keratinocytes are important in re-epithelialization and the restoration of the vascular network [6]. Clinical studies utilizing cultured autologous keratinocytes have demonstrated rapid re-epithelialization [14]. Additionally, sprayed autologous cells have shortened hospitalization times, reduced infection risk and yielded better functional and aesthetical outcomes [12,15,16,17]. Non-cultured epithelial cells can be immediately applied to the wound after biopsy isolation [18,19]. Techniques such as spraying cells necessitate the utilization of specialized technical devices to administer the cell suspensions. Cells can be delivered using an aerosol spray. One drawback associated with the use of cultured and non-cultured epithelial autografts is the absence of a dermal component, which is required to support the epidermal layer. However, cultured keratinocytes can be combined with a dermal substitute in the form of an allograft or synthetic mesh [16,20]. Keratinocytes can also be combined with tissue glue, such as fibrin [21]. The use of keratinocytes in burn injuries provides surgeons with a valuable tool for treating patients. However, it is imperative to establish a suitable spray delivery system. Currently, the available spray devices are either bulky or require compressed air or electricity, and may not be particularly easy to handle. Furthermore, the nozzle geometry of the delivery system used can affect the delivery of viable cells [22]. In this study, the biological effects of three different nozzles were compared to those of the commercially available Cell Spray applicator. Additional high-speed imaging series were conducted to characterize the liquid flow velocity when using different nozzles.

## 2. Materials and Methods

### 2.1. Nozzle Types

The Cell Spray applicator (Supplementary Material Figure S1A), provided by the German Institute for Cell and Tissue Replacement (DIZG gGmbH, Berlin, Germany), uses a 5 mL syringe filled with cell suspension. Airflow occurs via an electric pump, which filters the airflow through a 0.2 μm membrane to prevent contamination. This filtration eliminates airborne bacteria and other particles before they reach the nozzle once airflow commences. Once at the nozzle, the air and suspension mixture form a spray. The fixed airflow rate of this device is 2.4 L/min, with a fixed medium flow rate of 13 mL/min. Alternative nozzle one (AN1) is a commercially available air-assisted nozzle featuring an additional air reservoir port (Figure S1B). The airflow for this nozzle is powered by an electric pump equipped with an additional flow meter installed for regulating and monitoring the flow rate. Ambient air, before entering the nozzle, undergoes filtration through a 0.2 μm filter membrane. The nozzle can be combined with a Luer-Lock syringe. The second alternative nozzle (AN2) operates without airflow assistance (unassisted) and does not require an airflow system (Figure S1C). This unassisted nozzle can also be connected to a Luer-Lock syringe. Alternative nozzle three (AN3), also unassisted, comprises a single spray outlet positioned on the nozzle’s side, resulting in a 90-degree spray distribution angle (Figure S1D). This nozzle required an adapter for adjusting the application direction and was inserted in a Luer-Lock syringe. This adaptation ensured consistent conditions for all nozzles used at the various medium flow rates.

### 2.2. Spray Distribution Using A4

**Figure 1:**
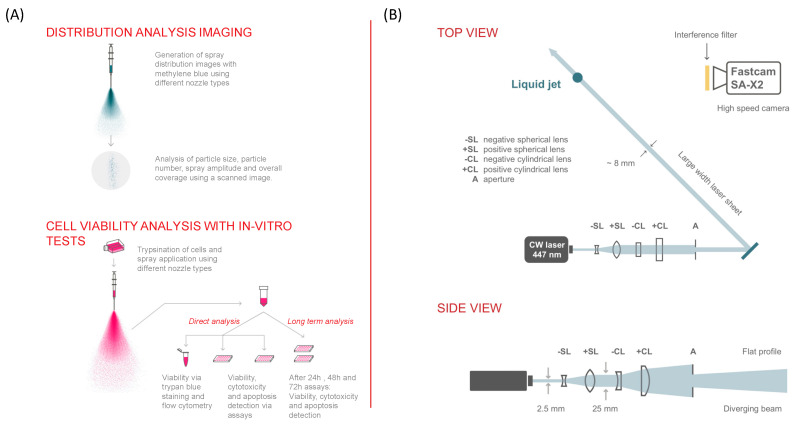
(**A**) Experimental setup for spray distribution image analysis and in vitro analysis. (**B**) Description of the experimental setup for imaging a large area (25 mm vertical distance) of the spray: a 447 nm, 3-Watt continuous wave laser was used as light source. Successive lenses allowed shaping the beam into a flat profile and generating a large diverging light sheet, which illuminated above 25 mm of the spray at 100 kHz frame rates. A single high-speed camera, Photron Fastcam SA-X2, was used for this experimental configuration. In addition, Phase Doppler Anemometry was used for droplet sizing measurements at 50 mm from the nozzle exit. Adapted from Ref. [23].

Different flow rates were used to visualize spray pattern and generate spray images for the different nozzles. This setup was designed to simulate clinical application conditions. Alternative nozzle one was tested using various air and medium flow rates, as shown in Table 1. The medium flow rates used for AN 2 and 3 were 2.5, 5, 7.5, 10, 13, 15, 20, and 30 mL/min. The applicator used a fixed airflow rate of 2.4 L/min and a medium flow rate of 13 mL/min. A material testing machine was used with a compression test setup. The syringe was positioned below the compression plate of the material testing machine. In a clinical setting, the cell sprayer is moved at a constant speed over the wound for complete coverage. Here, the spraying of cells used an altered agitator that was moved at a constant speed to mimic the clinical settings for wound coverage. An A4 sheet of paper was attached to the agitator’s rotation axis using a cord. With an axis diameter of 3.7 cm, the number of revolutions was adjusted to 103 rotations/min, resulting in a speed of 20 cm/min, which was estimated to correspond to clinical conditions. At the start of the recording, the paper was positioned vertically beneath the sample nozzle. A test medium consisting of cell-free 0.015% methylene blue solution was used to visualize the spray. The longitudinal distribution of the blue test medium, applied with specific flow rates through different nozzles, was recorded on the A4 paper (Figure 1A). The paper was then dried and scanned using a Kyocera Ecosys scanner (Meerbusch, Germany). Three replicates were generated for each nozzle and flow rate combination. All tubes, syringes, and materials used in the spraying process were sterile.

### 2.3. Spray Distribution Analysis of A4 Images

The number of particles, mean particle size, overall coverage, and spray amplitude were recorded. ImageJ (1.51.s, National Institute of Health, Bethesda, MD, USA) was used to analyze the scanned images. A pre-installed particle analyzer was used to study particle size and the number of spray droplets, and it classified particles according to their size. Self-designed macros were implemented to determine the spray amplitude and total area coverage. Spray amplitude was defined as 95% of the total blue-colored area. Area coverage was calculated as a percentage difference between colored and non-colored paper.

### 2.4. Extrusion Force

The constant extrusion rates were determined using a compression test with a material testing machine (Inspekt Table blue 10 kN, Hegewald & Peschke, Nossen, Germany). The syringe was secured on a stable support and placed beneath the compression plate of the machine. The medium flow rate was adjusted to a distance–time function to maintain a constant rate of compression. The force required to extrude the solution was measured using the material testing machine. The maximum and mean extrusion forces were analyzed. The tested medium flow rate and airflow rate were 13 mL/min and 2.4 L/min, respectively. This corresponds to the preset flow rate for the applicator device.

### 2.5. Cultivation of HaCaT Cells

In vitro assays were conducted to assess the viability, cytotoxicity, and apoptosis of HaCaT cells. The HaCaT cell line was obtained from the BCRT (Berliner Centrum für Reise- und Tropenmedizin), Berlin, Germany in 2014. The cells were stored in liquid nitrogen until use. The cells were passaged in T 175 cm^3^ cell culture flasks for in vitro testing. Adherent cells were washed with 10 mL PBS and incubated for 17 min at 37 °C with 10 mL 0.05% EDTA solution. Following aspiration of the EDTA solution, cells were detached with 5 mL 0.125% trypsin solution and incubated at 37 °C for 5–7 min. Trypsination was halted by adding 5 mL of medium to the flasks. The cell suspension was filtered through a 40-μm cell strainer to prevent cell aggregates. After centrifugation for 5 min at 300× *g*, the cells were resuspended in media and counted. A cell number of 3.5 million cells was seeded into T 175 cm^3^ flasks and cultured at 37 °C with 5% CO_2_ and Dulbecco’s Modified Eagle Medium (87%), sodium pyruvate (1%), penicillin-streptomycin (1%), L-glutamine (1%), and fetal bovine serum (10%). Approximately two days later, the cells were at 80–90% confluency and ready for the in vitro assays. Sterility was maintained through a combination of aseptic technique, antibiotics, and regular monitoring for contamination.

### 2.6. Cultivation of Human Donor Keratinocytes

Primary keratinocytes were isolated from human skin for clinical application and patient treatment. Residual keratinocytes, obtained with informed consent for research use, were stored at −80 °C and used for this study. Thawed samples were used for in vitro studies, with NIH-3T3 fibroblasts (DSMZ, Braunschweig, Germany) serving as feeder cells. In culturing primary human keratinocytes, the conventional approach as described by Rheinwald and Green utilizes lethally irradiated mouse fibroblasts (3T3 cells) as a feeder layer to promote keratinocyte adhesion and proliferation [9]. Once thawed, each vial was treated with 6 mL of enzyme stop medium. Keratinocytes were then centrifuged for 7 min at 200× *g*, while murine fibroblasts underwent centrifugation for 4 min at 200× *g*. Cells were resuspended, and their number was adjusted for a T 80 cm^3^ cell culture flask. A total of approximately 2–3 million keratinocytes and 1.5–2.5 million murine feeder cells were cultivated. Cells were cultured at 37 °C with 5% CO_2_ with Dulbecco’s Modified Eagle Medium, Hams F12, FBS, gentamicin, L-glutamine, insulin, choleratoxin, EGF, hydrocortisone, and sodium pyruvate. Feeder cells were removed by trypsination once a confluence of 50% was achieved. Subsequently, cells were washed with 10 mL PBS, the supernatant was removed, and fresh keratinocyte medium was introduced. At 85–90% confluency, in vitro studies were conducted. Confluent cells were washed with 10 mL PBS and trypsinized for 5–7 min with 5 mL of 0.125% trypsin solution. The reaction was stopped using 5 mL enzyme stop solution. Resuspended cells were then filtered through a 40-μm cell strainer for FACS analysis. Suspensions were centrifuged at 200× *g* for 7 min and resuspended in media. Cell numbers were determined using trypan blue staining and manually counted with a counting chamber. For the in vitro tests, 1 million cells/mL were used. Sterility was maintained with the use of gentamicin and continuous observation for contamination.

### 2.7. In Vitro Assays

Cells were transferred into single-use syringes and attached to the different nozzles. The syringes were filled with 1–2 mL of cell suspension and sealed with Luer stopper caps. The cells were then sprayed into 50 mL Falcon tubes using a material testing machine. Viable controls were not sprayed but were instead directly transferred into tubes. All tubes, syringes, and materials used in the spraying process were sterile. Spraying was performed in triplicate for each experimental group with either HaCaT cells or human donor keratinocytes. For human keratinocytes, an alternative nozzle was used in conjunction with the Cell Spray applicator. Three different flow rates were used (10, 20, and 30 mL/min). A 5 mL syringe was used for each flow rate. A viable control and a cytotoxicity control were included. For the cytotoxicity control, 0.1% Triton x-100 solution was mixed with keratinocytes. Cells were then sprayed at the three different flow rates in the same manner as the HaCaT cells.

Four assays were used in this study: the CellTiterGlo Luminescent Assay for viability, the CellTox Green assay for cytotoxicity, and the ApoToxGlo Triplex Assay for viability, cytotoxicity, and apoptosis. These assays are based on different biochemical mechanisms of action. The CellTiterGlo Luminescent Cell Viability Assay relies on the oxidation of luciferin to oxyluciferin, catalyzed by luciferase in the presence of magnesium ions, ATP, and molcular oxygen, which are only produced in viable cells. Oxyluciferin emits a luminescence signal, which can then be detected and quantified. The CellTox Green assay detects cytotoxicity by measuring the binding of CellTox Green to DNA released into the medium, indicating plasma membrane damage. Once bound, a fluorescent signal is generated.

The ApoToxGlo Triplex Assay is a combined assay. Cell viability is detected by a fluorescence signal created by the cleavage of GF-AFC to AFC by protease within the viable cell. Cytotoxicity is also detected by a fluorescence signal. The substrate bis-AAF-R110 is not able to enter live cells but is instead cleaved by the dead-cell protease to release R110, which generates a fluorescent signal. Viability and cytotoxicity are performed simultaneously as they do not affect each other, and measured using different filters for excitation and emission. The apoptosis test is set in the second step of the assay. It relies on the cleavage of a luminogenic substrate containing the DEVD sequence. Following this caspase-3/7 cleavage, a substrate for luciferase is released, resulting in the luciferase reaction and the production of light.

### 2.8. Viability via Trypan Blue Staining

The viability of the sprayed cell suspension was assessed using trypan blue staining. Cytotoxicity controls were performed in two ways: HaCaT cells were incubated with 25 μmol/L digitonin for 60 min, and primary cells were treated with Triton x-100 as a cytotoxicity control. The cells were then manually counted using the “Cell counter” software tool on ImageJ.

### 2.9. Viability via Flowcytometry Analysis

A volume of 100 μL of sprayed cell suspension was transferred to each well of a U-bottom 96-well plate and mixed with 150 μL of Live-Dead Staining Solution (Invitrogen, Waltham, MA, USA) per well. Cytotoxicity controls were prepared by incubating HaCaT cells with 25 μmol/L digitonin for 60 min. Primary cells used Triton x-100 as a cytotoxicity control. The plate was then incubated for 20 min at room temperature in the dark. Cell suspensions were analyzed with the BD Acuri Flow cytometer (BD Biosciences, San Jose, CA, USA) using the FL-4 channel.

### 2.10. Viability of HaCaT Cells

An ApoToxGlo Triplex Assay was used to determine the viability of HaCaT cells after spraying and was performed according to the manufacturer’s guidelines (Promega, Madison, WI, USA). Readouts were taken at 0, 24, and 48 h. Viability is detected using a fluorescence signal that measures GF-AFC cleavage to AFC by protease.

### 2.11. Viability of Donor Keratinocytes

A RealTime-Glo MT Cell Viability Assay and a CellTox Green Cytotoxicity Assay were used to identify cell viability and cell cytotoxicity for 72 h. These assays were conducted and multiplexed in accordance with the manufacturer’s guidelines (Promega, Madison, WI, USA). The RealTime-Glo assay utilizes the reducing potential of metabolically active cells. A prosubstrate as well as the corresponding luciferase are added to the culture medium. The prosubstrate enters the cells, is reduced to a substrate for the luciferase, and diffuses back into the surrounding medium. The enzyme then converts the substrate, thereby producing a luminescence signal that correlates with the number of metabolically active cells. The reactants of the kits were diluted 1:500 in the according pre-warmed medium and the assay was started by adding 50 μL of reaction mix to each well in order to reach a final dilution of 1:1000 of the reactants. The cells of the cytotoxicity control were sacrificed by adding 4 μL of Lysis Solution (CellTox Green Cytotoxicity Assay Kit) per well. The plate was incubated at 37 °C for 30 min. The fluorescence and luminescence signals were measured after 24, 48, and 72 h of culture using a microplate reader. The fluorescent dye was excited at 485 nm and the signal was measured with an emission filter of 528 nm.

### 2.12. High-Speed Imaging

Liquid-laser-induced fluorescence (LIF) was used to visualize the structure of the transparent liquid bodies in a spray, such as droplets, blobs, ligaments, and liquid core [23,24,25]. The liquid was pre-enriched with the fluorescent dye fluorescein at a concentration of $2.4\times 10^{4}\;mol\cdot L^{-1}$. Fluorescence is induced using a continuous wave laser at 447 nm with a maximum power of 3 W. To observe the general shape of the spray, the fluorescence signal was induced by a laser plane with a width of about 25 mm. The incident beam was shaped to obtain an illumination profile as flat as possible and to create a broad light sheet with a thickness of approximately 8 mm (the optical components and their respective arrangement are given in Figure 1B). Typically, sprays are visualized using shadowgraphy with a fast camera; in this study, LIF high-speed imaging was used to improve spray dynamic observations. Thus, the visualization was conducted with a high-speed camera (Photron SA-X2) running at 100,000 fps and placed in a backscattering configuration. The camera objective was a Nikon Micro-Nikkor with a 105 mm focal length operating at F# = 8. A high-performance fluorescence bandpass filter with a full width at half maximum of 89 nm, centered at 510 nm (Edmund Optics #84-113), was used to detect the fluorescence emission while rejecting the 447 nm scattered light. With this system, the resulting resolution was 38 µm per pixel. AN2 and AN3 were tested at three different injection pressures (high pressure “HP”, medium pressure “MP”, and low pressure “LP”) using the setup described in Figure 1B. To obtain information on the size of the droplets, Phase Doppler anemometry measurements (@532 nm) were performed at a distance of 30 mm from the nozzle outlet.

### 2.13. Statistical Methods

Graph Pad Prism 7 (GraphPad Software, Inc., San Diego, CA, USA) was used for the statistical analysis of in vitro tests. A two-way analysis of variance (ANOVA) was performed. Each treated cell mean was compared to the viable control mean. The Dunn’s t-test for multiple comparisons of the groups was linked to the ANOVA. Family-wise significance and the level of confidence were 0.05. For the multiplex assay, significance was calculated using RM 2-way ANOVA. Tukey’s multiple comparison test was used to calculate *p*-values. Significance is displayed as * *p* ≤ 0.05, ** *p* ≤ 0.01, *** *p* ≤ 0.001, and **** *p* ≤ 0.0001. A 95% confidence interval was used.

## 3. Results

### 3.1. Spray Particle Distribution

**Figure 2:**
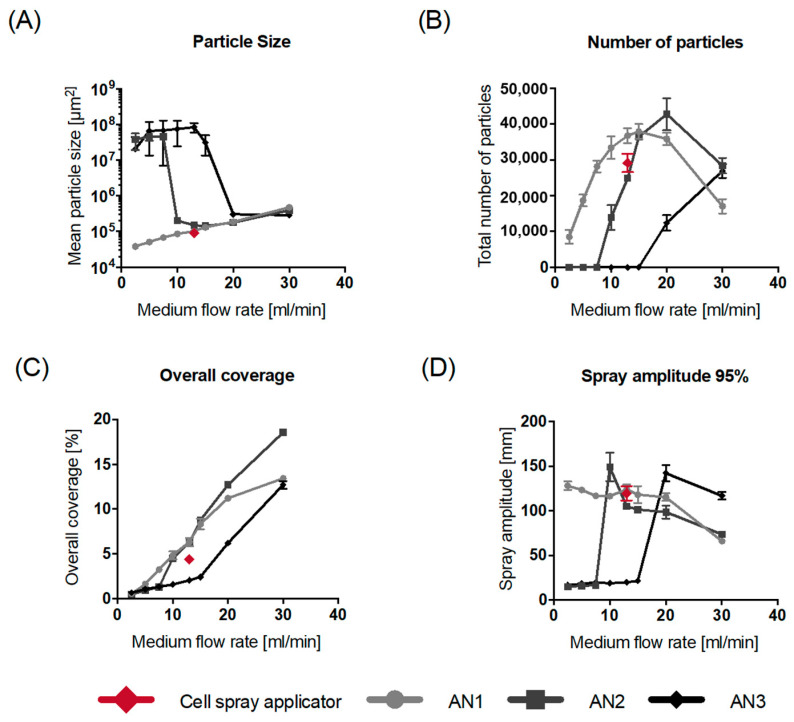
Different testing parameters for the four different nozzle types with varied medium flow rates. (**A**) Mean particle size. (**B**) Number of particles. (**C**) Overall coverage. (**D**) Spray amplitudes. Data are all displayed as mean values ± SD for *n* = 3 replicates. AN1 has a fixed airflow rate of 2.4 L/min in all figures.

The mean particle size was examined for the four distinct nozzles using a range of flow rates for both air and medium flow (Figure 2). AN1 generated small particles at all media flow rates. At a medium flow rate of 13 mL/min, the standard nozzle on the Cell Spray applicator produced the smallest mean particle size compared to AN1. The other nozzles produced drops until a certain medium flow rate was attained. The flow rates required for the different nozzles to generate small mean particle sizes were 10 mL/min and 20 mL/min for AN2 and AN3, respectively.

The number of particles from the different nozzles is depicted in Figure 2B. Interestingly, at the lower medium flow rates, AN1 exhibits high total particle counts. High medium flow rates of 20 and 30 mL/min were necessary to produce high particle numbers for the AN2 and AN3, respectively. Overall, among the tested nozzles, AN2 demonstrated the highest total particle count.

Furthermore. increasing the medium flow rate resulted in enhanced overall coverage for all nozzles (Figure 2C). The highest overall coverage was observed with AN2. Spray amplitude declined at medium flow rates of 20 mL/min or more (Figure 2D). Furthermore, the maximum extrusion force required to start spraying increased with elevated medium flow rates (Figure S2A). However, the increase in force was dependent on the nozzle used. The mean extrusion force was also analyzed for all the nozzles (Figure S2B) and varied depending on the medium flow rate and the nozzle type used.

In addition to the effects of the medium flow rate, the effect of airflow on particle size was investigated (Figure S3A). The smallest mean particle size was observed at lower airflow rates. The impact of airflow rate on overall coverage was also tested (Figure S3B). It was determined that when using a constant medium flow rate of 13 mL/min, different airflow rates did not influence the overall coverage. However, airflow rate affected the spray amplitude (Figure S3B) similarly to the effects of higher medium flow rates (Figure 2D).

### 3.2. In Vitro Assays

**Figure 3:**
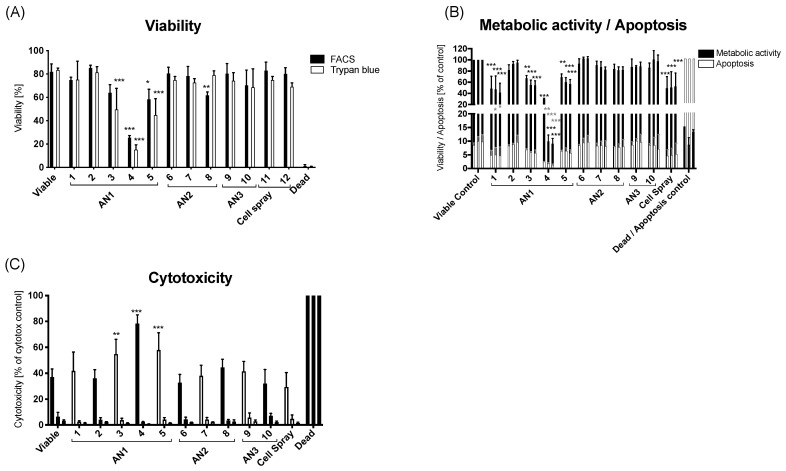
In vitro analysis of HaCaT cells for viability, cytotoxicity, and apoptosis. (**A**) Comparison of HaCaT viability via Flowcytometry and trypan blue staining with varied medium and airflow rates (Table 3). (**B**) Viability and apoptosis of sprayed HaCaT cells using the ApoToxGlo assay at T0, 24 h, and 48 h for each nozzle. Values are relative to the viable control (Table 3). Apoptosis values are relative to the apoptosis control, which was incubated with 10 μmol/L Staurosporine for 24 h (Table 3). Significance displayed in black corresponds to metabolic activity and grey corresponds to apoptosis. (**C**) Cytotoxicity of sprayed HaCaT cells using the ApoToxGlo assay at T0, 24 h, and 48 h for each nozzle (Table 3). Displayed is the mean value ± SD for *n* = 3 replicates. Significance is compared to controls. The different controls are all significant to the viable control with *p* < 0.001 for all time points for all figures. Dead cells were incubated with 25 µmol/L digitonin for 60 min. Significance is displayed as * *p* ≤ 0.05, ** *p* ≤ 0.01, *** *p* ≤ 0.001.

The viability of sprayed HaCaT cells was assessed using flow cytometry and trypan blue staining for each nozzle (Figure 3A). The air and medium flow rates are detailed in Table 2. A comparison of flow cytometry and trypan blue staining methods indicated comparable cell viability results. Notably, AN1 exhibited the most significant decrease in viability. Furthermore, an airflow rate of 4.2 L/min or greater significantly reduced cell viability, as evident in groups 3–5 (Figure 3A and Table 2). The most substantial reduction in cell viability was observed when using a low medium flow rate and a high airflow rate (group 4). The Cell Spray applicator with the standard nozzle was used first and last to establish that the chronological sequence of the nozzles and spray procedure did not impact viability (Figure 3A).

### 3.3. HaCaT Metabolic Activity, Apoptosis, and Cytotoxicity Using ApoToxGlo Triplex Assay

The metabolic activity and apoptosis of sprayed HaCaT cells were investigated for the various nozzles (Table 3) at three time points: T0 (cells still in suspension), 24 h, and 48 h (adherent cells) (Figure 3B). Overall, metabolic activity (Figure 3B) was relatively similar among groups except for the AN1 and the Cell Spray nozzle, which exhibited significantly lower values compared to the viable control. Apoptosis was also analyzed, revealing a significant decrease in group 4 compared to the viable control (Figure 3B). The cytotoxicity assay showed the highest values in group 4 at T0, correlating with the reduced viability shown in Figure 3A. Cytotoxicity then fell back to levels similar to other groups at 24 h and 48 h. This pattern was consistent across all samples, with T0 cytotoxicity elevated compared to later time points. Overall, higher airflow rates with air-assisted nozzles appear to negatively impact the HaCaT cells, particularly when combined with lower medium flow rates.

### 3.4. Keratinocyte Metabolic Activity and Cytotoxicity Using RealTime-Glo Assay and the CellTox Green Assay

**Figure 4:**
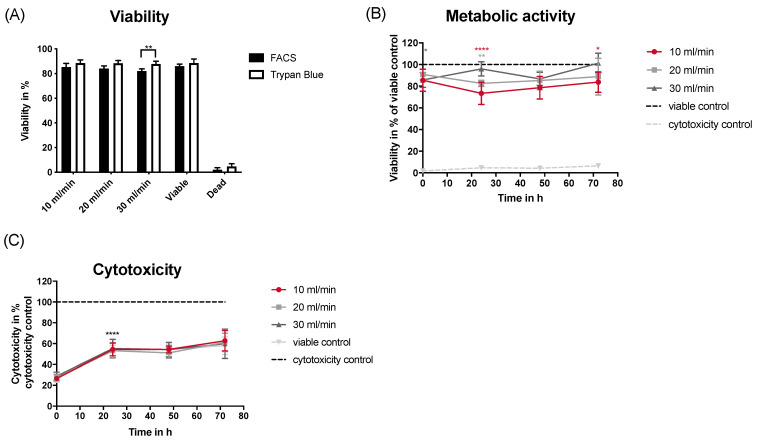
In vitro analysis of human donor keratinocytes for viability, cytotoxicity, and apoptosis. (**A**) Viability of sprayed donor keratinocytes via flow cytometry and trypan blue staining. Controls used were untreated cells as a viable control, and the dead control was achieved using 10% Triton x-100. (**B**) Viability of donor keratinocytes using the multiplex assay for 72 h after spraying. Controls used were untreated cells as a viable control and the cytotoxicity control was achieved using Lysis Solution. Luminescence was measured at 0, 24, 48, and 72 h after spraying. (**C**) Cytotoxicity of donor keratinocytes using the multiplex assay for 72 h after spraying. Controls used were untreated cells as a viable control and the cytotoxicity control was achieved using Lysis Solution. Fluorescence was measured at 0, 24, 48, and 72 h after spraying. Cells were all sprayed with varied flow rates of 10 mL/min, 20 mL/min, and 30 mL/min. Data shown as mean ± SD for *n* = 5 replicates (4 donors). Significance is displayed as * *p* ≤ 0.05, ** *p* ≤ 0.01, and **** *p* ≤ 0.0001. The colour of the significances correspond to the coloured lines.

Several crucial aspects for successful patient treatment using spray include small particle size, high particle count, and uniform coverage. Among the tested nozzles, AN2 emerged as the most suitable choice due to its performance in this study (particle number, size, and overall coverage). Additionally, AN2 is easy to operate because it lacks an airflow system. Consequently, the Cell Spray applicator was redesigned and paired with AN2 to investigate the delivery of primary human donor keratinocytes. Human donor keratinocytes were cultured for 72 h after spraying using AN2 at different flow rates (Figure 4). Multiplex analysis with the RealTime-Glo assay and the CellTox Green assay was used to assess the viability and cytotoxicity of keratinocytes over a 72 h period. In Figure 4A, the viability of human keratinocytes was directly investigated after spraying. Flow cytometry and trypan blue staining yielded comparable cell viability levels. A significant decrease was observed in the 30 mL/min group, with flow cytometry measurements indicating lower viability than trypan blue staining (*p* = 0.0087). However, this discrepancy might be attributable to trypan blue potentially underestimating cell viability [26].

Keratinocytes sprayed at a flow rate of 10 mL/min and 20 mL/min exhibited a significant decline in viability within the initial 24 h compared to the control (Figure 4B) (**** *p* ≤ 0.0001 and ** *p* = 0.0093, respectively). A flow rate of 20 mL/min did not demonstrate a statistically significant difference from the viable control, while a flow rate of 10 mL/min displayed a significant difference compared to that of the viable control (* *p* = 0.0176). Interestingly, cells sprayed at a rate of 30 mL/min initially showed significantly lower viability compared to the control, directly after spraying (* *p* = 0.0479). However, no significant difference was observed in viability at 72 h when using a 30 mL/min flow rate. In the cytotoxicity analysis, all groups produced similar results (Figure 4C). A significant rise in cytotoxicity was observed between T0 and 24 h after spraying (**** *p* ≤ 0.0001).

### 3.5. High-Speed Imaging

**Figure 5:**
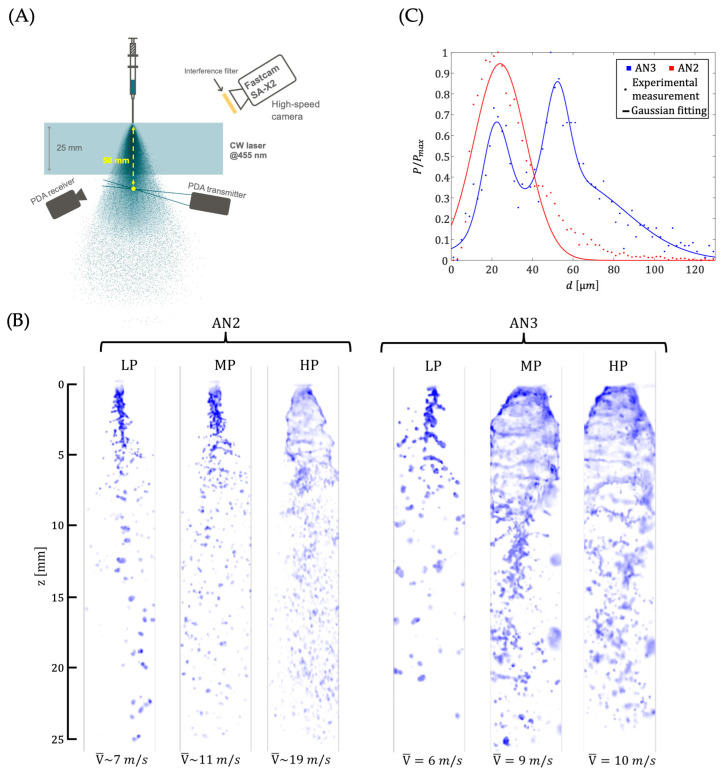
High-speed fluorescence imaging. (**A**) Experimental setup for the high-speed imaging experiments. (**B**) Images of the AN2 and AN3 nozzles for three different injection pressures (LP: low pressure, MP: medium pressure, and HP: high pressure). For each case, the mean droplet velocity is added in the bottom part of the images. Video of the sprays for the different conditions tested can be observed in Video S1. (**C**) Distribution of the droplet diameter in the case of the AN3 and AN2 injector for a high pressure. For each case, the resulting distribution is fitted by a Gaussian law.

Flow visualization [27] and droplet sizing measurements are generally carried out using Mie scattering measurement [28,29]. In this study, measurement of the nozzle was conducted using high-speed imaging and both unassisted nozzles, AN2 and AN3, were compared (Figure 5). This technique revealed that both nozzles required high pressure to achieve a well-defined spray structure with a clear atomization zone. This observation highlights the need for high-pressure operation when using these nozzles. At high pressures, AN3 generated a wider spray cone angle compared to AN2. This difference in spray cone geometry resulted in higher droplet velocities for AN2 (Video S1). The higher droplet velocity seems to be characterized by better atomization of the spray in the case of AN2 (higher Weber number). To further substantiate the notion that AN2 produces a more homogeneous spray with smaller droplets, phase-Doppler-anemometer (PDA, Artium instrument PDI-TK2) [30] measurements were conducted at high injection pressures for both AN2 and AN3. Figure 5B depicts the droplet diameter distribution normalized with the maximum value for AN2 and AN3 at high pressure. The results, fitted to a Gaussian distribution, demonstrate that AN2 generates smaller droplets compared to AN3. This finding further confirms the superior atomization capability of AN2. 

## 4. Discussion

Patients suffering from severe dermal disruptions, such as full- or partial-thickness burns, are prone to infections and excessive fluid loss. Restoring the skin’s barrier function, a process known as re-epithelialization, is crucial for healing. Keratinocytes, the primary epidermal cells, play a critical role in successful wound closure [6]. Cell transplantation, using either sheets or single-cell suspensions, can be performed, but the delivery system significantly impacts re-epithelialization efficiency [16]. Nozzle type is a critical determinant of cell spray performance. Studies have shown that decreasing nozzle diameter and increasing dispensing pressure can lead to mechanical damage of the cell membrane and loss of cell viability [31]. Additionally, an in silico nozzle design study informed by experiments has demonstrated that the nozzle geometry, such as nozzle radius, length, and material properties, can all influence cell viability, with shear stress used as a surrogate for cell survival [22]. The study presented here investigated the delivery of cell suspensions using commercially available nozzles. The tested nozzles exhibited varying effects on spray distribution and cell viability. Wound healing involves cell–cell interactions that drive keratinocyte migration and proliferation [32]. The applied keratinocytes determine the cell arrangement. Therefore, the spray distribution of a cell spray is expected to impact the homogeneity of the re-epithelialization process. A well-distributed cell suspension is crucial for efficient wound healing and should be carefully considered when designing a cell spray delivery system.

Small droplet size, high particle number, and ample surface coverage are key features of a well-distributed cell spray [33,34]. However, studies have shown that aerosol sprays operated at high air pressures negatively impact droplet size and distribution [35,36]. Here, particle size, particle number, and surface coverage were shown to depend on the medium flow rate and the nozzle used. For clinical use, spray generated at lower medium flow rates would offer benefits to both the patient and surgeon. By reducing the risk of large droplets carrying away cells, complete wound coverage could be achieved. This would enhance wound healing outcomes by promoting uniform cell distribution and preventing cell aggregation. This is important as the cultivation of keratinocytes takes a long time and only a limited number of cells are available. Additionally, smaller droplets would dry quicker on the wound, potentially penetrating deeper than larger drops and minimizing cell transfer and loss onto the wound dressing. Furthermore, higher particle numbers produced at lower flow rates would provide ample time for surgeons to administer the spray evenly over the entire wound area, ensuring wider coverage and minimal cell clumping.

Using air-assisted nozzles severely impacts cell viability and activity, with higher airflow rates leading to more pronounced reductions in cell survival, metabolic activity, and apoptosis, as shown in Figure 3B. A significant decrease in metabolic activity and increase in apoptosis were observed for the air-assisted nozzle. This would be in line with the effect that injection pressure can negatively affect viability due to mechanical damage to the cell membrane integrity, leading to a loss in cell viability [31]. In nozzle-based bioprinting, cell damage mainly comes from shear stress [37,38]. Shear stress on the cells can be transduced into a biological signal, ultimately leading to the activation of effector caspases [39]. Caspases are key regulators of cell death (apoptosis) [40] and the shear stress on cells may induce the apoptotic pathway. Veazey et al. [41] reported a viability of 37% when using a nozzle with a small outlet diameter and a high air pressure. In the case of AN1 group 4, the drastic reduction in metabolic activity and apoptosis may be due to the low initial number of viable cells added to the assay, leading to less metabolic activity and cell death (Figure 3A). In the case of the Cell Spray nozzle, a reduction in metabolic activity was observed. This can be explained by the assay measuring viability relative to the fluorescence of the viable control. This can be supported by the lack of a significantly strong cytotoxic signal in the Cell Spray group; however, this might be due to a lower number of initial cells being transferred. If the cell viability decreases, then it can be assumed that an increase in cytotoxicity or apoptosis occurs; however, this did not happen. The AN1 group 4 showed lower metabolic activity but also showed high cytotoxicity, highlighting that the higher airflow rate may have impacted or damaged the cells. Overall, it can be seen that airflow-assisted nozzles lead to lower metabolic activity and higher cytotoxicity levels. The apoptotic levels for all nozzles seemed to be under 10%. This is considered low and is supported by Amer et al. [42], who reported low apoptotic values for cells extruded with different needles. This has also been supported by Vernez et al. [43], who investigated apoptosis levels in cultured epidermal autografts before transplantation, reporting < 6% for the tested autografts. Immediately after spraying, all nozzles displayed a higher cytotoxic effect. This did not appear to continue over a longer period of time, suggesting that the cell membrane may experience shear stress upon spraying with higher airflow rates, displaying significantly higher cytotoxicity. Another important point regarding this assay is that the media is changed every 24 h, thus drastically reducing the cytotoxicity signal present in the medium. The media change will remove any DNA that was previously released into the culture after the cell damage, leading to a reduction in the assay signal. The level of cytotoxicity is of importance due to the possibility of affecting surrounding tissues by dispersing viable and damaged cells. Therefore, minimizing the cytotoxicity after spraying is of importance, especially in a clinical setting, and minimizing any potential harm to surrounding tissues is beneficial to the patients. The unassisted nozzle (AN2) performed very well regarding viability, apoptosis, cytotoxicity, particle size, and overall coverage. AN2 performed either similarly or better than the air-assisted nozzles. The success of AN2, the ability to connect it to a Luer Lock, and the lack of operational requirements such as an auxiliary air reservoir port, make it a suitable nozzle to use. Therefore, when AN2 was combined with the newly designed Cell Spray applicator, it displayed high viability of human donor keratinocytes post-spray, for all flow rates tested. Thus, lower delivery pressures do not affect the survival of donor keratinocytes when using AN2. Fredriksson et al. [44] compared different application techniques and observed similar results when using similar pressures. They observed a viability of 93.2% for the LINDAL Group nozzle, 84.1% for the Duploject nozzle and 76.6% for the Tissomat at low pressure. The Tissomat displayed lower viability (47.3%) at higher pressures [44]. Bovine fibroblasts were sprayed by Veazey et al. [41] at a pressure corresponding to a flow rate between 20 and 30 mL/min. In their study, viabilities of bovine fibroblasts varied from 86 to 94% depending on the nozzle diameter [41]. This further confirms what was observed with AN2. The real-time assay showed that in the initial 24 h post-spray, a decrease in viability was observed but recovered after 48 h. These findings are similar to that of Harkin et al. [35], who observed a 30% reduction in viability after 24 h compared to the viable control. Other studies have also shown recovery of cells after the initial 24 h [36,45]. The cytotoxicity data were generally higher than what was observed with HaCaT cells. This might be due to the nature of the real-time assay, where the medium is not changed over the period tested. As observed in this study, low-pressure devices seem to lead to better cell survival [44,46]. Patients would benefit from this as cell function will be preserved and more viable cells would reach the wound bed, promoting successful grafting. The lower cytotoxicity will also minimize potential harm to surrounding tissues.

Furthermore, the maximum and mean extrusion rates displayed by all the nozzles were well within the maximum human force required to press a syringe. A previous study investigated the forces needed to use a syringe with different needles [47]. Cilurzo et al. [47] observed that forces of up to 250 MPa made injection practically impossible, whereas forces lower than 125 MPa led to smooth injection. The needle diameter used in this study was 0.6 mm; therefore, the values can be converted into extrusion forces. A force of 250 MPa is equivalent to 70.75 N, with 160 MPa equaling 45.28 N and 125 MPa equaling 35.37 N. When compared to the study by Cilurzo et al. [47], all extrusion forces were within the applicable range. During the spraying procedure, a pressure sensor was included to measure the resistance. The pressure sensor recorded an extrusion force ranging from 2–11 N, with most values ranging between 2 and 5 N (Figure S2).

Several factors influence cell viability and spray capability during the spraying procedure. Extrusion force is crucial and a certain force is needed for proper atomization to achieve a good cone-shaped spray distribution. However, excessively high forces lead to poor atomization, resulting in large, non-uniform droplets instead. Lower forces are ideal for optimal spray formation. Furthermore, high extrusion forces compromise cell viability by increasing shear stress on cells as they pass through the nozzle [31]. Dispensing pressure, constant pressure rate, and nozzle diameter can also all influence cell viability [22]. Studies have shown that high cell viscosity and velocity negatively affect viability [36]. Extrusion force and nozzle geometry impact droplet size, which in turn affects cell viability. Smaller droplets tend to position cells in the center, where shear stress is lower compared to the edges of the spreading film [36,48]. Additionally, high droplet velocity can reduce viability due to impact-induced damage [36]. Numerous factors play a role in cell viability and functionality during spraying. Identifying parameters that minimize these negative effects is crucial.

High-speed imaging provides a valuable tool for use in the medical field. Spray analysis using high-speed imaging demonstrated good spray distribution with an upside-down tulip shape that was apparent when using AN2. Alternative nozzle 3 displayed large droplet sizes compared to AN2 at the same pressure. Moreover, a homogeneous spray pattern was observed for AN2 at higher pressures. Here, high-speed imaging confirms that even when using AN2 at high or maximum pressures, a uniform spread of cells can still be achieved with high viability, as demonstrated with the previously used flow rates. It can also be noted that the AN3 nozzle displayed a bi-modal distribution of the droplet diameter while AN2 displayed a mono-modal distribution. For patient treatment, a mono-modal distribution of sprayed cells would be ideal to maintain an evenly spread healing process.

## 5. Conclusions

This study reports that unassisted nozzles provide high cell viability, good metabolic activity, and spray distribution at different medium flow rates and pressures. Thus, unassisted nozzles are suitable for covering a wound bed and for restoring the skin’s barrier function in the case of burn injuries. The delivery system used to transfer cells to burn wounds can directly impact the cells’ survival and, in turn, the re-epithelization process, preventing efficient healing. Thus, it is important to use a high-quality manufactured nozzle from a certified manufacturer to provide surgeons with a treatment device that can deliver cells and cover the wound site without harming the keratinocytes, leading to successful treatment of the patient. This newly designed device provides surgeons with a simple and easy-to-use applicator, omitting the need for an airflow system. This can lead to a straightforward implementation and reduces the risk of contamination due to the lack of airflow filters.

## Figures and Tables

**Table 1:** Air and medium flow rates used for the generation of spray distribution data.

Airflow Rate in L/min	Medium Flow Rate in mL/min
2.4	2.5
2.4	5
2.4	7.5
2.4	10
2.4	13
2.4	15
2.4	20
2.4	30
2.4	13
3	13
4	13
6	13

**Table 2:** The different medium and airflow rates used with the different nozzles to identify the viability of sprayed HaCaT cells.

No.	1	2	3	4	5	6	7	8	9	10	11	12
Nozzle type	AN1					AN2			AN3		Cell sprayer	
											Start	End
Medium flow rate [mL/min]	2.5	30	16.25	2.5	30	10	20	30	20	30	13	13
Airflow rate [L/min]	2.4	2.4	4.2	6	6	-	-	-	-	-	2.4	2.4

**Table 3:** The different medium and airflow rates used with the different nozzles to identify the metabolic activity and apoptosis, and cytotoxicity of sprayed HaCaT cells.

No.	1	2	3	4	5	6	7	8	9	10
Nozzle type	AN1					AN2			AN3	
Medium flow rate [mL/min]	2.5	30	16.25	2.5	30	10	20	30	20	30
Airflow rate [L/min]	2.4	2.4	4.2	6	6	-	-	-	-	-

## Data Availability

The raw data supporting the conclusions of this article will be made available by the authors on request.

## Supplementary Materials

The following supporting information can be downloaded at: https://www.mdpi.com/article/10.3390/jfb15050126/s1, Figure S1: The different nozzles used. (A) DIZG Cell Spray. (B) Air-assisted nozzle, AN1. (C) Unassisted nozzle, AN2. (D) Unassisted nozzle, AN3; Figure S2: (A) Maximum extrusion force of the different nozzles using a variety of medium flow rates. (B) Mean extrusion force for the different nozzle types using a variety of medium flow rates. Data are all displayed as mean value ± SD for *n* = 3 replicates. Figure S3: Varied airflow testing of AN1. (A) Size and Number of particles achieved by AN1 using a fixed medium flow rate of 13 mL/min and a variety of airflow rates. (B) Spray amplitude and overall coverage using AN1. A constant medium flow rate of 13 mL/min and a variety of airflow rates are used. Data are all displayed as mean value + SD for *n* = 3 replicates. Video S1: Video of high-speed fluorescence spray with nozzle comparison.
