# Supplementary material for: High-Speed Fluorescence Imaging Corroborates Biological Data on the Influence of Different Nozzle Types on Cell Spray Viability and Formation
Source: J Funct Biomater. 2024 May 14;15(5):126. doi: 10.3390/jfb15050126 (PMC11122036; doi:10.3390/jfb15050126)
Supplement: Supplementary file 1 [file jfb-15-00126-s001.zip › jfb-2974102-1st proof - supple.pdf]

Journal of Functional Biomaterials

Supplementary Materials

High-Speed Fluorescence Imaging Corroborates Biological Data on the Influence of Different Nozzle Types on Cell Spray Viability and Formation

Miriam Heuer [1], Mehdi Stiti [2], Volker Eras [1], Julia Scholz [1], Norus Ahmed [1,*], Edouard Berrocal [2] and Jan C. Brune [1]

[1] German Institute for Cell and Tissue Replacement (DIZG, Gemeinnützige GmbH), Haus 42, Köpenicker Str. 325, 12555 Berlin, Germany; m_heuer@dizg.de (M.H.); v_eras@dizg.de (V.E.); j_scholz@dizg.de (J.S.); j_brune@dizg.de (J.C.B.)
[2] Division of Combustion Physics, Department of Physics, Lund University, P.O. Box 118, 22100 Lund, Sweden; mehdi.stiti@imft.fr (M.S.); edouard.berrocal@fysik.lu.se (E.B.)
* Correspondence: n_ahmed@dizg.de

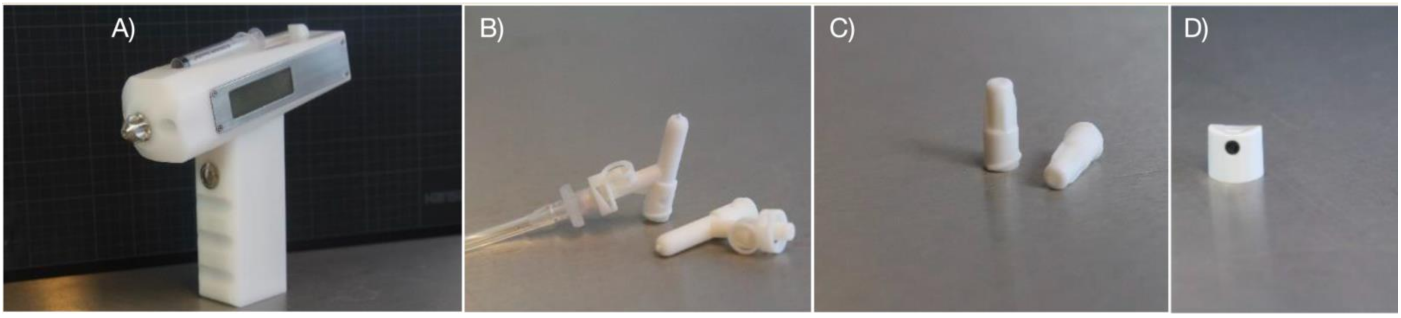

Figure S1. The different nozzles used. (**A**) DIZG Cell Spray. (**B**) Air-assisted nozzle, AN1. (**C**) Unassisted nozzle, AN2. (**D**) Unassisted nozzle, AN3.

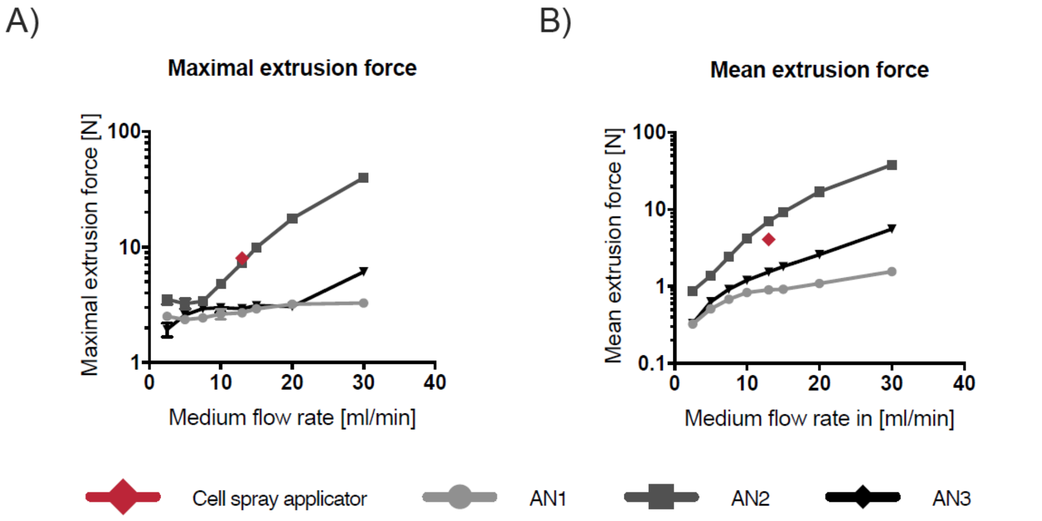

Figure S2. (**A**) Maximum extrusion force of the different nozzles using a variety of medium flow rates. (**B**) Mean extrusion force for the different nozzle types using a variety of medium flow rates. Data are all displayed as mean value ± SD for $n = 3$ replicates.

Citation: Heuer, M.; Stiti, M.; Eras, V.; Scholz, J.; Ahmed, N.; Berrocal, E.; Brune, J.C. High-Speed Fluorescence Imaging Corroborates Biological Data on the Influence of Different Nozzle Types on Cell Spray Viability and Formation. *J. Funct. Biomater.* **2024**, *15*, 126. https://doi.org/10.3390/jfb15050126

Academic Editor: Chunming Wang

Received: 4 April 2024
Revised: 29 April 2024
Accepted: 09 May 2024
Published: 24 May 2024

Copyright: © 2024 by the authors. Licensee MDPI, Basel, Switzerland. This article is an open access article distributed under the terms and conditions of the Creative Commons Attribution (CC BY) license (https://creativecommons.org/licenses/by/4.0/).

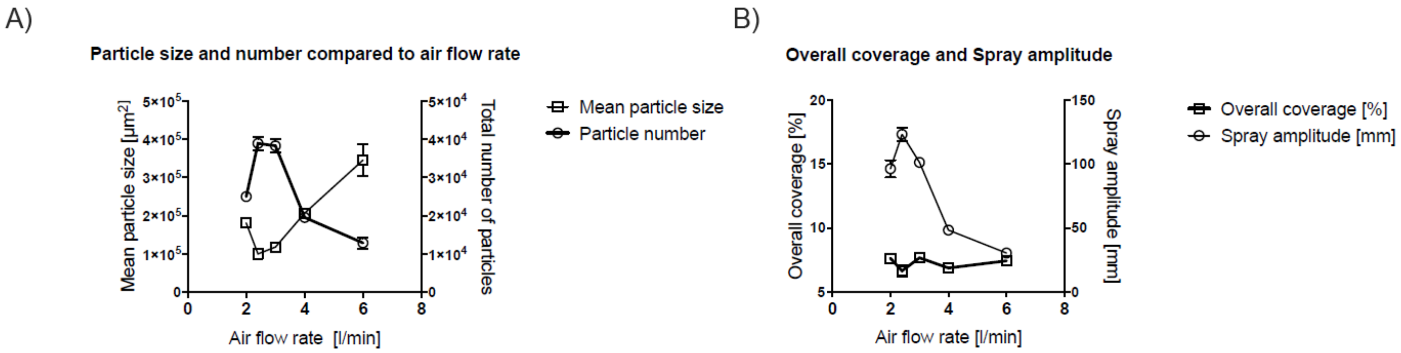

Figure S3. Varied airflow testing of AN1. (**A**) Size and Number of particles achieved by AN1 using a fixed medium flow rate of 13 mL/min and a variety of airflow rates. (**B**) Spray amplitude and overall coverage using AN1. A constant medium flow rate of 13 mL/min and a variety of airflow rates are used. Data are all displayed as mean value + SD for n = 3 replicates. .

Disclaimer/Publisher's Note: The statements, opinions and data contained in all publications are solely those of the individual author(s) and contributor(s) and not of MDPI and/or the editor(s). MDPI and/or the editor(s) disclaim responsibility for any injury to people or property resulting from any ideas, methods, instructions or products referred to in the content.
